# Spontaneous gallbladder perforation in a man with gallstone disease without known anamnesis of cholecystitis: Case report

**DOI:** 10.1016/j.ijscr.2023.108731

**Published:** 2023-09-03

**Authors:** Pål Ødegaard, Ljiljana Blecic-Johansen, Jeanette Cooper, Airazat M. Kazaryan

**Affiliations:** a Department of Surgery, Østfold Hospital Trust, Grålum, Norway; b Department of Gastrointestinal and Pediatric Surgery, Oslo University Hospital - Ullevål, Oslo, Norway; c Institute for Clinical Medicine, Medical Faculty, University of Oslo, Oslo, Norway; d Department of Surgery, Fonna Hospital Trust, Odda, Norway; e Department of Faculty Surgery № 2, I.M. Sechenov First Moscow State Medical University, Moscow, Russia; f Department of Surgery № 1, Yerevan State Medical University after M. Heratsi, Yerevan, Armenia

**Keywords:** Spontaneous gallbladder perforation, Acute abdomen, Challenging diagnosis

## Abstract

**Introduction:**

Spontaneous gallbladder perforation is a rare complication of gallstone disease. It causes acute peritonitis with potentially fatal outcome.

**Case presentation:**

We present a case of spontaneous gallbladder perforation with challenging diagnosis.

**Discussion:**

The diagnosis of gallbladder perforation should be considered in elderly patients presenting to the surgical emergency department with symptoms and signs of peritonitis even in the absence of pre-existing gallbladder disease. Spontaneous gallbladder perforation is a rare and potentially fatal diagnosis. In most reported cases, perforation occurred due to predisposing factors like acute cholecystitis, trauma or obstruction. In spite of rarity, spontaneous gallbladder perforation should be considered as differential diagnosis on examination of patients with sudden abdominal pain especially in cases of known cholecystolithiasis. Our patient had type 1 perforation according to Niemeier classification, the type associated with the highest mortality rate. The type of perforations has been reported as being difficult to recognize preoperatively, as with our patient with two inconclusive CT scans. This was due to the absence of classical symptoms of gallbladder perforation. CT is the modality of choice when gallbladder perforation is suspected.

**Conclusion:**

We believe the reason for the spontaneous gallbladder perforation in the presented case was the presence of cholecystolithiasis. We acknowledge the importance of considering this diagnosis also in patients without previous signs of cholecystitis.

## Highlights

• The case presents a spontaneous gallbladder perforation, an uncommonly encountered diagnosis in the surgical emergency department.
• The patient had no predisposing factors.
• Challenges in establishing of correct diagnosis is pointed out.

## 1 Introduction

Acute abdominal pain is one of the most common causes for patient referral to a surgical emergency department (ED). Gallbladder perforation occurs as a complication of acute cholecystitis in 2–11 % of patients [1,2]. We present a rare case of spontaneous gallbladder perforation without prior history of cholecystitis.

## 2 Case presentation

This clinical case is reported in line with the SCARE criteria [3]. A 65-year-old man presented to the ED with 3 h history with an episode of sudden severe abdominal pain attack. It was initially located in the right hypochondrium, migrating to the epigastric region. It was associated with nausea and vomiting. The patient had no significant comorbidity, prior history of gallstone disease, and no history of previous abdominal surgery. On arrival to the ED the blood pressure was 120 / 77 mmHg, pulse 77 beats/min, temperature 36.7 °C, oxygen blood saturations (SpO2) of 97 % with 3 L oxygen, respiration frequency 17 breaths/min. The examination revealed guarding and epigastric tenderness. The biochemistry showed C-reactive protein (CRP) at 5 mg/L, with blood cells (WBC) elevated at 16.7 10^9^ /L, potassium 3.4 mmol/L, bilirubin 13 μmol/L, aspartate aminotransferase 32 U/L, alanine transaminase 44 U/L, gamma-glutamyltransferase 36 U/L, amylase serum 15 U/L. Arterial blood gas showed pH 7.3, pCO_2_ 5.5 kPa, pO_2_ 11.7 kPa, bicarbonate 22 nmol/L, BE −3.7 nmol/L and lactic acid 4 mmol/L.

**Fig. 1:**
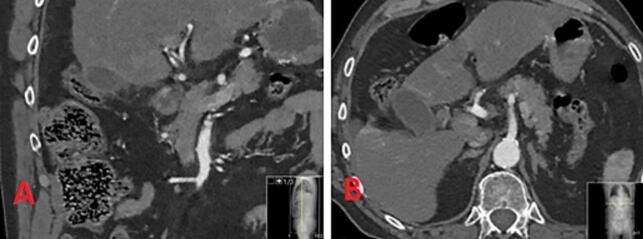
Computed tomography of the chest and the abdomen with an aorta angiography protocol (day 1): A. Frontal view; B. Transverse view.

At first due to sudden manifestation, a ruptured abdominal aortic aneurysm was suspected, and the patient was taken to CT (computed tomography) of the chest and the abdomen with the aorta angiography protocol (Fig. 1). Early acute pancreatitis and perforated ulcer were also included in the differential diagnoses. CT showed no sign of thoracic or abdominal aneurysm, no free fluid or gas in the abdomen, no sign of intestinal obstruction, but gallbladder stones without signs of acute cholecystitis and no signs of biliary hypertension. Extensive colon diverticula without signs of diverticulitis was revealed. Due to the extensiveness of the colon diverticula, and the now more diffuse location of pain, a tentative diagnosis of diverticulitis was made; intravenous antibiotics were started and the patient was admitted for close observation at the surgical department.

**Fig. 2:**
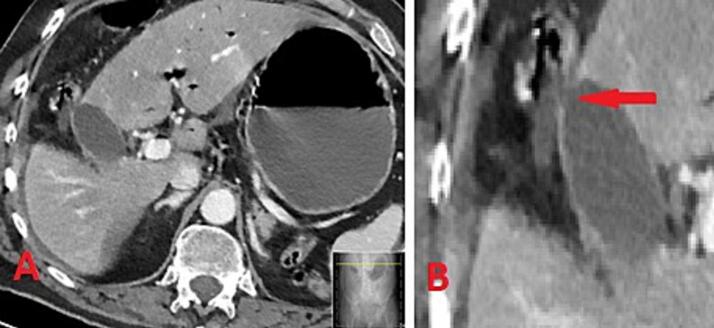
Computed tomography with the abdomen (day 2): A. Frontal view; B. Magnification of the image with an arrow showing perforation (verified after retrospective scrutinization after surgery).

The next day, CRP increased to 300 mg/L and WBC decreased to 8.7 10E9/L. Abdominal examination revealed increased distention of the abdomen and diffused tenderness. The hemodynamics worsened with development of tachycardia (pulse 124 beats/min) and tendency to hypotension (blood pressure 117/86 mmHg). Body temperature was registered on 38.4 °C, SpO_2_ 93 % at 6 L of oxygen and respiration frequency of 20 breaths/min. Due to uncertainty around the diagnosis, a new abdominal CT scan was ordered, which showed intraluminal air in the portal and mesenteric veins, distended small bowels with air/fluid levels, as well as suspected mechanical intestinal obstruction and ventricle retention and free fluid in the abdomen, no signs of biliary hypertension (Fig. 2).

**Fig. 3:**
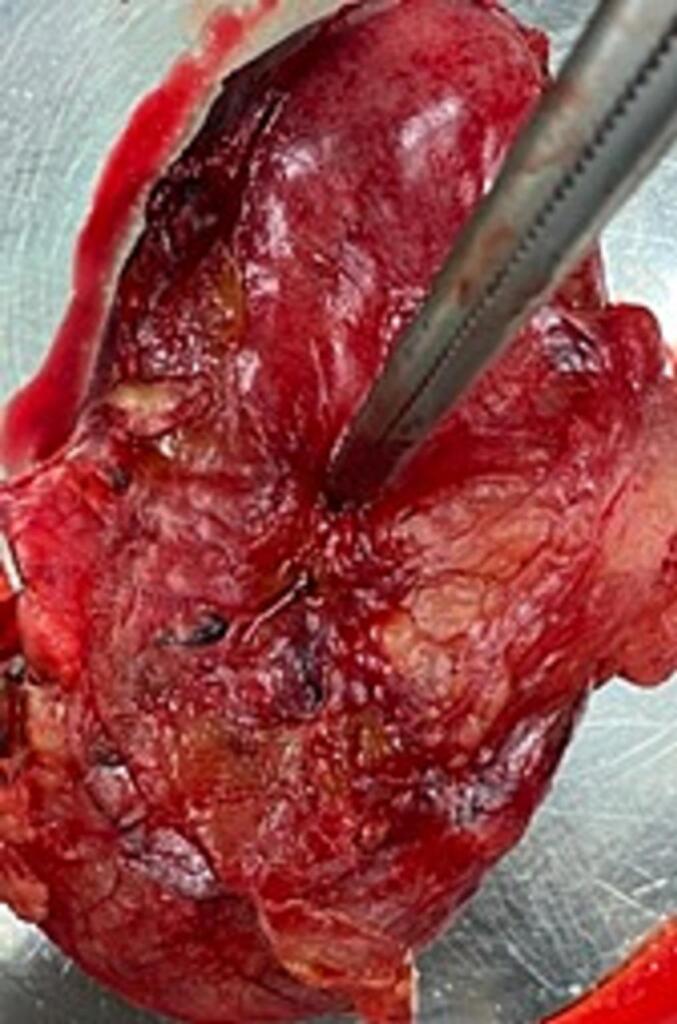
Removed gallbladder. The forceps inserted in the gallbladder perforation.

Indications for surgical exploration of the abdomen was considered as absolute and emergent midline laparotomy was performed, which immediately revealed over 1 L of bilious fluid and dilated small bowels. Full intraoperative inspection was done which revealed a 2 mm hole in the inferior peritoneal surface of the gallbladder body (Fig. 3). Open cholecystectomy and lavage of the abdominal cavity were performed. Due to hemodynamically unstable patient, laparotomy opening was covered by a conditioning vacuum packing, and a secondary closure of the abdomen was performed 48 h later. Histopathological investigation showed gallbladder with chronic inflammation with an area of acute inflammation and perforation.

## 3 Discussion

The diagnosis of gallbladder perforation should be considered in elderly patients presenting to the surgical emergency department with symptoms and signs of peritonitis even in the absence of pre-existing gallbladder disease. Gallbladder perforation occur in approximately 0.8–18% [1,2] of patients with acute cholecystitis, and is usually due to delay in presentation or start in treatment.

Spontaneous gallbladder perforation is a rare and potentially dangerous diagnosis with reported mortality of 12–42% [4]. In most reported cases, perforation occurred due to predisposing factors like acute cholecystitis, trauma or obstruction [5]. In spite of rarity, spontaneous gallbladder perforation should be considered as differential diagnosis on examination of patients with sudden abdominal pain especially in cases of known cholecystolithiasis. According to Niemeier classification [6] of gallbladder perforations, they divide into three subtypes (see Table 1).

**Table 1:** Niemeier classification of gallbladder perforation.

Type	State	Description
Type 1	Acute	Free perforation and biliary peritonitis
Type 2	Subacute	Fluid localized at site of perforation, with local peritonitis and abscess formation.
Type 3	Chronic	Include formation of fistula with adjacent organs.

Type 2 is the most common (46.2 %), followed by type 1 (40.6 %) and type 3 (10.1 %), with most common cause for gallbladder perforation being cholelithiasis (86.6 %) [4].

Although this classification, elaborated in 1934, was criticized due to being founded on purely clinicopathological findings [4], there is no other widely accepted classification scale. Estevão-Costa [5] proposed a new classification system, which classifies the perforations into three types: - 1: spontaneous, 2: traumatic and 3: iatrogenic. Group 1 is further subdivided into idiopathic and secondary, and group 2 subdivided into penetrating and blunt trauma.

Our patient had type 1 perforation according to Niemeier classification, the type associated with the highest mortality rate [5]. The type 1 has been reported as being difficult to recognize preoperatively [7,8], as with our patient with two inconclusive CT scans. This was due to the absence of classical symptoms of gallbladder perforation. The gallbladder fundus is reported as most common location of gallbladder perforation (60 %) [1,2,7]. Studies have shown that CT is superior to ultrasonography in detecting the gallbladder rupture [9], and the extensive use of radiology and CT scans in diagnosis of acute abdomen have made the diagnosis easier. CT is the modality of choice when gallbladder perforation is suspected, and magnetic resonance has not been widely used in the emergency setting, even though its capabilities are considered to be superior to CT [10].

## 4 Conclusion

We believe the reason for the spontaneous gallbladder perforation in the presented case was the presence of cholecystolithiasis. We acknowledge the importance of considering this diagnosis also in patients without previous signs of cholecystitis.

## Guarantor

Yes, all authors.

## Research registration number

Not applicable.

## Consent

Written informed consent was obtained from the patient to publish this report in accordance with the journal's patient consent policy.

## Ethical approval

A written consent was received from the patient. In such case, the anonymised presentation of case report does not require a separate approvement by the ethics committee.

## Funding

No specific funding. The study was performed as a part of clinical / academic positions of the coauthors.

## CRediT authorship contribution statement

PØ: Manuscript draft and revision.

LBJ: Supervision and revision.

JC: Revision.

AMK: Idea, supervision and revision.

## Declaration of competing interest

None declared.
